# Circadian Clock Genes in the Metabolism of Non-alcoholic Fatty Liver Disease

**DOI:** 10.3389/fphys.2019.00423

**Published:** 2019-05-08

**Authors:** Dongmei Shi, Jie Chen, Jiaofeng Wang, Jianfeng Yao, Yiqin Huang, Gansheng Zhang, Zhijun Bao

**Affiliations:** ^1^Department of Gastroenterology, Huadong Hospital, Fudan University, Shanghai, China; ^2^Department of Geriatrics, Shanghai Key Laboratory of Clinical Geriatric Medicine, Huadong Hospital, Fudan University, Shanghai, China

**Keywords:** circadian rhythm, circadian clock, non-alcoholic fatty liver disease (NAFLD), circadian clock gene, metabolism

## Abstract

Non-alcoholic fatty liver disease (NAFLD) is a common disease, which is characterized by the accumulation of triglycerides in the hepatocytes without excess alcohol intake. Circadian rhythms can participate in lipid, glucose, and cholesterol metabolism and are closely related to metabolism seen in this disease. Circadian clock genes can modulate liver lipid metabolism. Desynchrony of circadian rhythms and the influences imparted by external environmental stimuli can increase morbidity. By contrast, synchronizing circadian rhythms can help to alleviate the metabolic disturbance seen in NAFLD. In this review, we have discussed the current research connections that exist between the circadian clock and the metabolism of NAFLD, and we have specifically focused on the key circadian clock genes, Bmal1, Clock, Rev-Erbs, Rors, Pers, Crys, Nocturnin, and DECs.

## Introduction

Non-alcoholic fatty liver disease (NAFLD) is a form of triglyceride (TG) accumulation at or exceeding 5% of the liver weight without excess alcohol intake (Andronescu et al., 2018). The histological classification distinguishes a range of conditions within NAFLD that vary from hepatic steatosis to non-alcoholic steatohepatitis (NASH), which might evolve to many subsequent conditions that include fibrosis, cirrhosis, liver failure, and hepatocellular carcinoma (Ipsen et al., 2018).

The global prevalence of NAFLD has dramatically increased during the past three decades, as a result of a global epidemic in the incidence of metabolic disorders. The current prevalence rates of NAFLD vary from 17 to 51% in western countries and 25% in Asian countries (Stern and Castera, 2017; Wong et al., 2018). In China, the prevalence varies from 15 to 30%.

In our study, we have revealed that the prevalence of NAFLD in employees of the city of Shanghai, China was 38.17%; a rate that was much higher than we had previously appreciated (Hu et al., 2012; Sherif et al., 2016). Furthermore, it was reported that the prevalence of NAFLD in children is increasing, and although putative mechanisms have received broad discussion, biological reasons accounting for the prevalence and contributions to NAFLD remain an enigma (Friedman et al., 2018).

**FIGURE 1:**
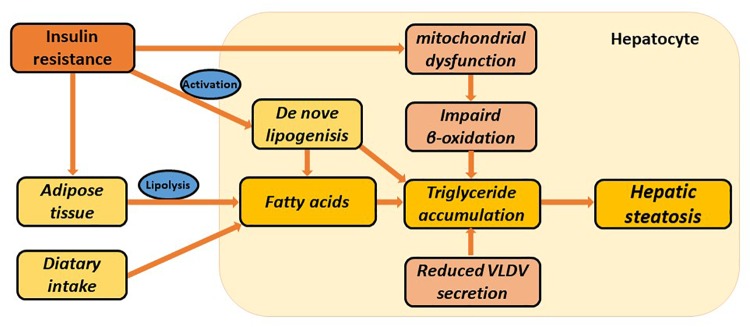
Multiple metabolic disorders involved in the pathogenesis of hepatic steatosis. Hepatic steatosis can be stimulated via increased adipose tissue lipolysis, increased *de novo* lipogenesis, increased dietary fatty acid uptake, impaired β-oxidation, and reduced VLDL synthesis and secretion. Insulin resistance is particularly involved in several metabolic pathways. These all lead to hepatic triglyceride accumulation and ultimately NAFLD.

Multiple factors lead to abnormal accumulation of triglycerides (TGs) in hepatocytes, among which, insulin resistance (IR) is the most essential pathogenesis. The role of insulin in inhibiting the decomposition of fat cells is weakened where IR is seen, resulting in lipolysis from the adipose tissue, and increased uptake of free fatty acids into the liver. At the same time, the utilization of TGs by the liver is inhibited, which provokes lipid deposition in the liver (Asrih and Jornayvaz, 2015). Fat accumulation in the liver can be traced by an increased incidence of *de novo* lipogenesis. In addition, mitochondrial dysfunction could impair fatty acid beta-oxidation and cause lipid accumulation, which usually precedes NAFLD. Excessive TG is transported out of the cell by binding to intra-hepatic synthesis of VLDL (Ipsen et al., 2018). With impaired fatty acid beta-oxidation or TG transport, the capacity of the liver to clear lipids efficiently is reduced, which might ultimately lead to the development of NAFLD (Figure 1).

Circadian rhythms in mammals are hard-wired biological systems that follow the 24-h cycle of the day, which serve to precisely regulate many of the major physiological activities, including sleep/wake cycles, feeding/fasting cycles, endocrine rhythms and of course metabolic rhythms (Bass and Takahashi, 2010). Studies provide a strong link between circadian rhythm disruption and the onset of a variety of human diseases. Desynchrony of circadian rhythms and the external environment, such as shift work, chronic jet lag, intentional sleep restriction, deprivation and night eating can markedly contribute to increased morbidity. Shift workers for example, exhibit a higher prevalence of obesity and associated disorders, such as NAFLD (Johnston, 2014). By contrast, restoration of normal circadian rhythms can improve overall health and alleviate the observed morbidity (Oosterman et al., 2015). The circadian rhythm system thus plays a key role in human physiology and disease systems.

A connection between the circadian rhythm and NAFLD has relatively recently been proposed (Gnocchi et al., 2015). Circadian rhythms are closely related to metabolic diseases and can participate in lipid, glucose, and cholesterol metabolism. Resynchronizing circadian rhythms can help alleviate this observed metabolic disturbance (Mazzoccoli et al., 2018).

In this review, we will discuss current research connections between the circadian clock and the metabolism seen in NAFLD, with a particular focus on key circadian clock genes.

## The Molecular Network of Circadian Clock

**FIGURE 2:**
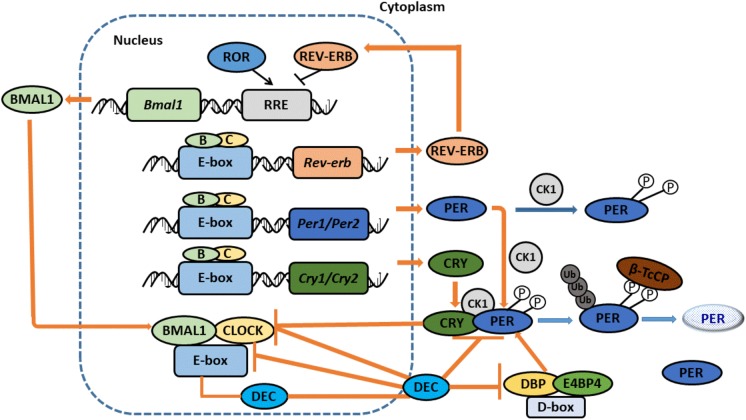
The Molecular Network of Circadian clock. CLOCK and BMAL1 dimerize to stimulate the expression of CCGs with E-box promoter. CLOCK: BMAL1 also activate the expression of the PERS and CRYS. When PERs and CRYs protein levels become high, they dimerize and translocate to the nucleus to repress CLOCK: BMAL1-mediated transcription. PERs and CRYs undergo post-translational modifications that induce their degradation, prepared a new circadian cycle. Proteins REV-ERBα/b, whose levels increase during the day, bind specific responsive promoter elements (RRE) and inhibit BMAL1 transcription. At night, REV-ERBα proteins levels are low, allowing BMAL1’s transcription. DECs also can form an additional loop. DECs repress their own transcription by directly binding to BMAL1 and/or E-box sites. DECs can also repress the transcription of PER1 and DBP. Furthermore, DBP and E4BP4 can bind D-box, activate and suppress transcription to stabilize and fine-tune the PER/CRY feedback loop, and thus promote the formation of the fourth loop. Adopted with modification from Masao Doi.

The molecular circadian clock network is composed of four transcriptional-translational feedback loops. The core loop, which includes Clock (circadian locomotor output cycles kaput) and Bmal1 (brain and muscle-ARNT-like 1, also known as ARNTL1), generates the autonomous circadian rhythm (Yoo et al., 2017) (Figure 2). Clock and Bmal1 heterodimerize and induce the transcription of clock-controlled genes (CCGs) in a process that involves binding with E-box (5′-CACGTG-3′). The heterodimers also direct the transcription of their functional repressors that include period (Per1, 2, and 3) and cryptochrome (Cry1 and 2), with the ultimate formation of a self-regulated loop (Gerber et al., 2015). When the protein expression levels of Pers and Crys achieve a high level, they dimerize and translocate to the nucleus to repress Clock: Bmal1-mediated transcription. Pers and Crys undergo post-translational modifications that induce their degradation in biological readiness for a new circadian cycle. In the second loop, Clock: Bmal1 heterodimers regulate the transcription of the DNA-binding orphan nuclear receptor reverse erythroblastosis virus-α/β (Rev-Erb-α/β, Rev-Erbs), and the retinoid-related orphan receptor-α, -β, and -γ (Ror-α, -β, -γ, and Rors). Rors activate, while Rev-Erbs repress, the transcriptional expression of Bmal1 (Orozco-Solis and Sassone-Corsi, 2014). The Rev-Erbα/b proteins, whose levels increase during the day, bind specific responsive promoter elements (RRE) and inhibit Bmal1 transcription (Guillaumond et al., 2005). At night, Rev-Erbα protein levels are low, allowing for the transcription of Bmal1. Recently, the expression of DECs, which include DEC1 (Bhlhe40/Stra13/Sharp2) and DEC2 (Bhlhe41/Sharp1) have been found to function as clock genes that can form the third autonomous feedback loop. DECs can repress their own transcription by binding to Bmal1 or by binding to E-box sites by competing with Clock: Bmal1 (Sato et al., 2018). DECs can also repress the transcription of Per1 and DBP (albumin d-element binding protein) (Kawamoto et al., 2004). Furthermore, DBP and E4BP4 can bind D-box, activate and suppress transcription to stabilize and fine-tune the Per/Cry feedback loop, and thus promote the formation of the fourth loop (Yamajuku et al., 2011). In the liver, the sex of animals is a pivotal regulator of circadian clock genes that include Rev-Erbα, Rorγ, and Cry1 (Doi, 2012).

## Circadian Clock Genes and Metabolism in the Setting of Nafld

NAFLD is essentially an imbalance of lipogenesis between free fatty acid (FFA) supply, formation, utilization, and disposal. The circadian clock is also closely associated with metabolism. Circadian clock genes tightly modulate the metabolism of liver lipids, and disruption of the biological circadian rhythms induces lipid accumulation in the liver (Mazzoccoli et al., 2014; Shi et al., 2014). However, the exact mechanisms remain obscure and unresolved at this time. Targeting the clock genes in the mouse model will benefit our improved understanding of how circadian rhythms interact with a variety of metabolic disorders (Sahar and Sassone-Corsi, 2012) (Table 1).

**Table 1:** The metabolism and mechanism in clock gene mutant mice.

	Lipid relatedmetabolism	Glucose relatedmetabolism	Other functions	Mechanism
**BMAL1**				
Bmal1^-/-^ mice	Developing hepatic steatosis in the regular chow-feeding. reduced fat storage, increased circulating fatty acids, increased ectopic fat formation in liver and muscle.	Glucose intolerance, hypoinsulinemia	Up-regulation of Bmal1 mouse lipid synthesis activity in adipocytes embryonic fibroblast lacking Bmal1 are unable to differentiate into adipocytes.	Bmal1: Clock can act with SREBP-1c, FASN, HMGCR, ELOVL, LDLr, AACS to control the daily lipid metabolism in the liver. Bmal1 can function as a cAMP-responsive coactivator of HDAC5 to regulate hepatic gluconeogenesis. Bmal1 can drive PPARs transcription while PPARs in their turn activate the transcription of Bmal1. Furthermore, in the mouse liver, Bmal1 expression can be repressed KLF. KLF10 can sense intracellular glucose levels and bind two juxtaposed GC boxed which are near the Bmal1 gene transcription promoting site. Bmal1 regulates the 24-h periodicity expression of KLF10, which in the mouse liver modulates the circadian genes variation in lipogenesis, gluconeogenesis, and glycolysis.
Liver-specific Bmal1^-/-^ mice	Dyslipidemia, an elevation of circulating FFA, high hepatic TGs.	Hypoglycaemia during fasting. Greater glucose clearance despite normal insulin production.		
Pancreas-specific Bmal1^-/-^ mice	Normal adiposity.	Severe glucose intolerance, similar to whole-body Bmal1^-/-^ mice.	Intact behavioral circadian rhythms.	
Skeletal muscle-specific Bmal1^-/-^ mice		Insulin-stimulated glucose uptake impaired in muscle, fasting blood glucose and glucose toleranceare normal.		
Adipose-specific Bmal1^-/-^ mice	Increased weight gain and adipose tissue mass, with more food consumption during the daytime.			
Heart-specific Bmal1^--/-^ mice		Systemic insulin resistance and decreased insulin-induced phosphorylation of AKT in the liver.	Decreased heart function.	
Tissue-specific Bmal1^-/-^ mice	Not showing ectopic fat formation fed with HFD	An age-dependent reduction in size, with elevated ROS levels.	Germline overexpression of Bmal2 can rescue Bmal1^-/-^ mice. Muscle-specific overexpression of Bmal1 can rescue the low body weight and early death phenotypes of Bmal1 knockout mice.	
Global and liver-specific Bmal1^-/-^ and APOE^-^/^-^ mice	Increased hyperlipidemia and atherosclerosis.		Overexpression of Bmal1 can reduce hyperlipidemia and atherosclerosis.	
**CLOCK**				
Clock^-/-^ mice	Reduced hepatic triglyceride accumulation with HFD.			
Clock mutant mice	Obviously higher TG in the liverwith HFD			
Clock^Δ19/Δ19^ double-mutant mice	Hepatic steatosis, obesity, hypertriglyceridaemia, increased absorbing lipids throughout the day. The levels of plasma triglyceride are high all times. Other aberrant expressions of metabolism-regulating genes have also been observed, including FABP1, ACS4, HMGCR, LDLr.	Hyperglycaemia		Reduced expression of Pdia3, which can bind the E-box motif and transcriptionally regulated by Bmal1:Clock. Inhibiting Pdia3 can activate the PERK pathway and induce the activation of oxidative stress and apoptosis.
Clock^Δ19/Δ19^ ApoE^-/-^ and Clock^Δ19/Δ19^ LDLr^-/-^ mice	Higher atherosclerosis. such mice can assemble and secrete more chylomicrons and have higherlipid levels.		Macrophages of Clock^Δ19/Δ19^ ApoE^-/-^ mice are defective in cholesterol efflux.	
**NPAS2**				
NPAS2^-/-^ mice			NAPS2 can sense cellular metabolic state. adapting slowly to restricted feeding.	
NPAS2 deficiency in SHP^-/-^ mice	Severe hepatic steatosis because of lipoprotein metabolic derangement.			NPAS2 can bind to the SHP promoter rhythmically and promote its circadian expression with elevating levels of NAD+.
**REV-ERBs**				
Rev-Erbα/HDAC3^-/-^ mice	Hepatic steatosis			Rev-Erbα can modulate liver lipid metabolism by epigenetic changes induced recruiting HDAC3, subsequently with chromatin remodeling and histone modification.
Liver-specific HDAC3^-^/^-^ mice	Severe hepatic steatosis associated with increased *de novo* lipogenesis.	Higher insulin sensitivity		Low Rev-Erbα levels reduce HDAC3 association with the liver genome during the activity/feeding time and permit lipid lipogenesis. Elevated Rev-Erbα levels enhance HDAC3 recruitment to liver metabolic genes in the resting/fasting time, hindering lipid lipogenesis.
Rev-Erbα^-/-^ mice	Elevated VLDL levels and increased APOC-III expression in the liver.			Rev-Erbα can promote circadian signaling via INSIG2–SREBP and LXR, which participants in bile acid and lipid metabolism.
Both liver Rev-Erbα and Rev-Erbβ deficient mice	Remarkable hepatic steatosis		Rev-Erb agonists can treat the circadian lipidome. Rev-Erb treatment can induce weight loss and decrease plasma TGs, cholesterol and fatty acids in mice.	
**RORs**				
The Rorα mutant mouse (RORα^sg/sg^, staggerer mouse)	Reduced body fat, smaller fat cells in brown and white adipose tissue, lower liver TGs levels, less susceptible to hepatic steatosis, but severe atherosclerosis though fed with more food.			Rorα and its ligands can induce the expressions of SOD2 and GPx1, reduce hepatic oxidative stress and inflammation reaction, and alleviate NASH in mice. Rorα can enhance M2 polarization in liver macrophages which protects hepatocytes from injury by secreting IL-10.
Myeloid-specific knockout of Rorα	Enhancing the liver susceptibility toHFD-induced NASH.			Rorα activator induces M2 polarity switch in Kupffer cells and protects the liver progressing to NASH.
**PERs**				
Whole body Per2^---^ mice	Lowered levels of TGs and non-esterified fatty acid. In white adipose tissue, TG is reduced while levels of saturated and monounsaturated very-long-chain fatty acidsare elevated.			
Both Per1 and Per2 deficient mice		Impaired glucose tolerance.		Through histone H3 acetylation, the promoter regions of Per1 and Per2 undergo circadian fluctuation. Per2 can particularly regulate lipid metabolism by directly blocking PPARα, PPARγ, and Rev-Erbα transcription in white adipose and liver tissue.
Per3	Regulating the clock of APC and modulating adipogenesis *in vivo*. Deleting Per3 promotes adipogenesis.		AMPK, a cell sensitive sensor of low energy and nutrient state, can manage the degradation of Per and CYR proteins.	Per3 and Bmal1 can directly regulate KLF15 expression.
**CRYs**				
CRYS		Through acting on G protein-coupled receptor signaling, CRYs can regulate hepatic gluconeogenesis, block cAMP accumulation and activate the transcription of gluconeogenic genes regulated by CREB.		Crys link the circadian clock, JAK and JAK-signal transducer and STAT signaling through regulating STAT5B phosphorylation. Crys can also repress genes transcription encoding the glucocorticoid receptor and phosphoenol pyruvate carboxykinase. Autophagy can degrade Cry1 and regulate the liver clock and glucose metabolism through controlling LIR motifs.
Cry deficient mice			Smaller body and organ size.	
Cry1/Cry2 double knockout mice	Abnormal TGs levels in the serum and the liver.	Glucose intolerance.	Chronically elevated circulating corticosterone levels with augmented glucocorticoid-dependent transactivation in the liver. showing an additional metabolic phenotype, salt-sensitive hypertension.	
In diabetic mice	HFD can accelerate the degradation of Cry1 and induce to obesity-associated hyperglycemia.	Liver-specific overexpression of Cry1 can lower blood glucose and increase insulin sensitivity.		
**NOCTURNIN**				
Nocturnin	Directly involving in lipid absorption, regulating unknown reduced lipid trafficking in the small intestine. participating in adipogenesis.	Participating in glucose homeostasis.	Participating in osteogenesis and immune function.	Encoding a deadenylase involved in the removal of polyA tails from mRNAs.
Nocturnin^-/-^ mice	Displaying resistance to HFD-induced obesity and hepatic steatosis. During the daily circadian cycle and acute nutritional challenges, having markedly elevated metabolism of cholesterol and TG.		Having normal circadian mechanisms.	
**DECs**				
DECs	DEC1 and DEC2 can regulate adipogenesis by repressing the transcription of PPARγ. Overexpressing of DEC1 suppress adipocyte differentiation.	Insulin and glucose induce DEC1 and DEC2 expression, depleting glucose decreases DEC1 and DEC2 expression. mammalian target of rapamycin can inhibit insulin-induced DEC1 and DEC2 expression.		Which encode bHLH transcription factors, can regulate the circadian rhythm and metabolism. DEC1 also can interact with DNA-bound CCAAT/enhancer binding protein and repress PPARγ expression.
DEC1^-/-^ mice	Decreased lipid levels		Reduced oxidative stresses, and increased FGF21 levels.	
DEC2^-/-^ mice				In liver, pAMPK is remarkedly increased. LXR can induce DEC1 expression by binding its promoter. Blocking phosphoinositide 3-kinase, PK C.

### Bmal1

Bmal1 plays an important role in the modulation of fat storage, utilization and adipocyte differentiation. Up-regulation of Bmal1 increases lipid synthesis activity in adipocytes (Tong and Yin, 2013). Bmal1^-/-^ knock-out mice show glucose intolerance, hypoinsulinemia, reduced fat storage, increased circulating fatty acids, increased ectopic fat formation in the liver and muscle, and hepatic steatosis even with regular chow-feeding (McDearmon et al., 2006; Landgraf et al., 2017). Liver-specific Bmal1^-/-^ knock-out mice exhibit hypoglycemia during fasting, and greater glucose clearance despite normal insulin production and dyslipidemia, including high circulating FFA levels and high hepatic TGs (Pan et al., 2016). Pancreas-specific Bmal1^-/-^ knockout mice show severe glucose intolerance, despite intact behavioral circadian rhythms and normal adiposity. Skeletal muscle-specific Bmal1^-/-^ knockout mice show impaired insulin-stimulated glucose uptake in the muscles, while fasting blood glucose and glucose tolerance activities appear normal. Adipose-specific Bmal1^-/-^ knockout mice show increased weight gain and adipose tissue mass, which might be due to increased food consumption during the daytime hours. Heart-specific Bmal1^-/-^ knockout mice show decreased heart function, systemic insulin resistance and decreased insulin-induced AKT phosphorylation in the liver (Nakao et al., 2018). By contrast, global and liver-specific Bmal1^-/-^ knockout and APOE^-/-^ knockout mice show increased hyperlipidemia and atherosclerosis. Hepatic overexpression of Bmal1 in liver-specific Bmal1^-/-^ knockout and APOE^-/-^ knockout mice can reduce hyperlipidemia and atherosclerosis. Mouse embryonic fibroblasts lacking Bmal1 cannot differentiate into adipocytes (Grechez-Cassiau et al., 2008). Furthermore, tissue-specific (i.e., liver, skeletal muscle, fat, bone, spleen, kidney, testis, heart, and lung) Bmal1^-/-^ knockout mice show an age-dependent reduction in size, and do so consistently with elevated levels of ROS (Nakahata et al., 2018). Germline overexpression of Bmal2 can rescue Bmal1^-/-^ knockout mice (Reinke and Asher, 2016). Muscle-specific overexpression of Bmal1 can rescue low body weight and early death phenotypes that are seen in Bmal1 knockout mice (Gooley, 2016).

Bmal1: Clock cooperates with SREBP-1c, and downstream genes like fatty acid synthase (FASN), 3-hydroxy-3-methylglutaryl-CoA reductase (HMGCR), and fatty acid elongase family members (ELOVL), the low-density lipoprotein receptor (LDLr), and acetoacetyl-CoA synthetase (AACS) to modulate daily lipid metabolism in the liver (Friedrichs et al., 2018). Bmal1 can function as a cAMP-responsive coactivator of HDAC5 to regulate hepatic gluconeogenesis (Li et al., 2018). Bmal1 can drive PPARs transcription, while in turn, PPARs can activate the transcription of Bmal1, which drives PPARα and activates PPARα targeted gene expression. PPARα can directly bind PPAR response elements (PPRE), which are located upstream of the transcription initiation site in the Bmal1 promoter where they serve to regulate Bmal1 expression (Matsusue et al., 2014). PPARγ positively acts on Bmal1 expression by binding the same PPRE site (Pettersson-Klein et al., 2018). Further, in the mouse liver, Bmal1 expression is transcriptionally repressed by the member of three-zinc finger family of Kruppel-like transcription factors (KLF). KLF10 (also known as TGFb inducible early gene-1, or TIEG1) can sense intracellular glucose levels and bind two juxtaposed GC boxes that spatially located near the Bmal1 gene transcription promotor site. Bmal1 regulates the 24-h periodic expression of KLF10, which in the mouse liver, modulates variations in circadian gene expression in lipogenesis, gluconeogenesis, and glycolysis. Reciprocal control between PPARα/Bmal1, PPARγ/Bmal1 and KLF10/Bmal1 provides fine metabolic regulation in accord with the circadian rhythms system (Guillaumond et al., 2010).

Interestingly, sex dimorphism can modulate the acrophase of hepatic Bmal1, and can do so because of differential androgenic and estrogenic hormonal circadian regulation. The daily expression of hepatic Bmal1 in lean male mice (LM) is similar to that found in other studies. Acrophase occurs during the transition from dark to light cycles in LM, while in lean female mice (LF), acrophase is found during the transition from light to dark (Pérez-Mendoza et al., 2018). Increased lipogenic gene expression is observed in obese males with reduced levels of hepatic Bmal1 are found at the end of the light phase, while the same observations were found in Bmal1^-/-^ knockout mice (Soeda et al., 2017).

### Clock

Similarly, Clock is closely associated with NAFLD. Clock^-/-^ knockout mice display a reduced hepatic triglyceride accumulation under HFD conditions. Clock mutant mice with HFD show a clearly increased TG in the liver. Contrary to observations seen in wild-type mice, Clock^Δ19/Δ19^ double-mutant mice, are characterized by hepatic steatosis, obesity, hypertriglyceridemia and hyperglycemia, and show increased absorption of lipids throughout the day. The levels of plasma triglyceride in Clock^Δ19/Δ19^ double-mutant mice do not show circadian rhythms and are high at all times of the day. Other aberrant expressions of metabolism-regulating genes have also been observed, including fatty acid binding protein1 (FABP1), acyl-CoA synthetase 4 (ACS4), HMGCR, and of the low-density lipoprotein receptor (LDLr) (Pérez-Mendoza et al., 2014). Clock^Δ19/Δ19^ double-mutant mice show reduced expression of Pdia3, which can bind the E-box motif, which is transcriptionally regulated by Bmal1: Clock. Inhibiting Pdia3 can activate the PERK-mediated signaling pathway and induces activation of oxidative stress and apoptosis. In addition, the PERK signaling pathway, appears to be the key pathway that is predominantly regulated by Clock.

Clock^Δ19/Δ19^ ApoE^-/-^ knockout and Clock^Δ19/Δ19^ LDLr^-/-^ knockout mice show higher levels of incidences of atherosclerosis. Physiologic studies indicate that such mice assemble and secrete more chylomicrons and have higher lipid levels. Moreover, macrophages of Clock^Δ19/Δ1^ ApoE^-/-^ knockout mice are defective in cholesterol efflux (Sookoian et al., 2008).

Neuronal PAS domain-containing protein 2 (Npas2), paralogous to Clock, is mainly expressed in the brain with lower levels in the peripheral tissues, while Clock is mainly expressed in the peripheral tissues (Reick et al., 2001). Naps2 can also sense the cellular metabolic state. Most likely due to the function of Clock in SCN neurons, loss of Npas2 does not affect feeding patterns or weight gain. Npas2^-/-^ knockout mice adapt slowly to restricted feeding (Schleicher et al., 2015). The orphan nuclear receptor small heterodimer partner (SHP) can inhibit Npas2 gene transcription and promoter activity, while Npas2 can bind to the SHP promoter rhythmically and promote its circadian expression while elevating levels of NAD+. Npas2 deficiency in SHP^-/-^ knockout mice can lead to severe hepatic steatosis because of disrupted lipoprotein metabolism (Lee et al., 2015).

### Rev-Erbs and Rors

Rev-Erbα and its close homolog Rev-Erbβ (also known as nuclear receptor subfamily1, group D, member 1; NR1D1) are heme-dependent transcriptional repressors, whereas Ror -α, -β, and -γ (Rors) are transcriptional activators. Rev-Erbs and Rors can recognize the same DNA binding sites (Ror response elements, RREs) and perform opposing regulatory biological functions (Hu et al., 2012).

### Rev-Erbs

Rev-Erbα can epigenetically modulate liver lipid metabolism that subsequently recruits histone deacetylase 3 (HDAC3), and induces chromatin remodeling and histone modification. Rev-Erbα/HDAC3^-/-^ knockout mice display hepatic steatosis. Liver-specific HDAC3^-/-^ knockout mice show severe hepatic steatosis that is associated with increased *de novo* lipogenesis and higher insulin sensitivity. Low Rev-Erbα levels reduce the capacity for HDAC3 to associate with the liver genome during the time of activity/feeding, which permits lipid lipogenesis. By contrast, elevated Rev-Erbα levels enhance HDAC3 recruitment to liver metabolic genes during the resting/fasting time, hindering lipid lipogenesis. Rev-Erbα can promote circadian signaling via the INSIG2–SREBP pathway and the liver X receptor (LXR), which participates in bile acid and lipid metabolism. Rev-Erbα^-/-^ knockout mice show elevated VLDL levels and increased APOC-III expression in the liver. Both liver Rev-Erbα and Rev-Erbβ deficient mice display remarkable hepatic steatosis (Fathi Dizaji, 2018). Rev-Erb agonists have been used to treat the circadian lipidome. Beyond altering circadian behavioral patterns and clock gene expression, Rev-Erb treatment can induce weight loss and decrease the levels of plasma TGs, cholesterol and fatty acids in mice (Liu et al., 2008).

### Rors

Rorα and its ligands can induce the expression of SOD2 and GPx1, reduce hepatic oxidative stress and inflammatory reactions, and can alleviate NASH in mice (Monnier et al., 2018). The Rorα mutant mouse (Rorα^sg/sg^; i.e., the staggerer mouse), though fed with increased quantities of food, has reduced levels of body fat, visibly smaller fat cells in the brown and white adipose tissues, and lower liver TGs levels, as well as decreased susceptibility to hepatic steatosis, contrasted by evidence of severe atherosclerosis (Solt et al., 2012). Moreover, a prior published study found that Rorα can enhance M2 polarization in liver macrophages, which protects hepatocytes from injury by secreting the anti-inflammatory and immuno-modulatory cytokine IL-10. Myeloid-specific knockout of Rorα enhances liver susceptibility to HFD-induced NASH. In addition, the Rorα activator induces an M2 polarity switch in Kuepfer cells and protects the liver progressing to NASH (Han et al., 2014, 2017).

### Pers

Through histone H3 acetylation, the promoter regions of Per1 and Per2 undergo circadian fluctuation. Beyond binding core clock genes, Per2 can particularly regulate lipid metabolism by directly blocking PPARα, PPARγ, and Rev-Erbα transcription in white adipose and liver tissue. Whole body Per2^-/-^ knockout mice showed decreased levels of TGs and non-esterified fatty acids. Compared to wild-type mice, white adipose tissue, and TG is reduced, while levels of saturated and mono-unsaturated very-long-chain fatty acids are elevated in Per2^-/-^ knockout mice (Grimaldi et al., 2010). AMPK, which is a cell sensitive sensor of a low energetic and nutritional state, manages the degradation of expressed Per and CYR proteins. Both Per1 and Per2 deficient mice show impaired glucose tolerance. Per3 can regulate the clock of adipocyte precursor cells (APC), and can modulate adipogenesis *in vivo*. In addition, both Per3 and Bmal1 can directly regulate KLF15 expression. By contrast, deleting Per3 expression can promote adipogenesis.

### Crys

Crys link the circadian clock, Janus kinase (JAK) and JAK-signal transducer and activator of transcription (STAT) signaling by regulating STAT5B phosphorylation. By their action on G protein-coupled receptor signaling, Crys can regulate hepatic gluconeogenesis, block cAMP accumulation and activate the transcription of gluconeogenic genes that are regulated by CREB (Zhang et al., 2010). Crys can also repress gene transcription that encode the glucocorticoid receptor and phosphoenol pyruvate carboxykinase. Glucocorticoids induce Per2 expression and affect glucose metabolism under hyperglycemic conditions. In addition, Cry deficient mice have smaller body and organ sizes. Cry1/Cry2 double knockout mice show abnormal TGs levels in the serum and liver, glucose intolerance and chronically elevated circulating corticosterone levels with augmented glucocorticoid-dependent transactivation in the liver. Furthermore, Cry1/Cry2 double knockout mice display an additional metabolic phenotype of salt-sensitive hypertension (Chaudhari et al., 2017). In diabetic mice, liver-specific overexpression of Cry1 can lower blood glucose and increase insulin sensitivity. HFD can accelerate the degradation of Cry1 and induce obesity-associated hyperglycemia. Autophagic pathways can degrade Cry1 and regulate the liver clock and glucose metabolism by modulating the expression of light chain3 (LC3)-interacting region (LIR) motifs (Toledo et al., 2018).

### Nocturnin

Nocturnin (also known as Noct, formerly known as Ccrn4l) displays a vigorous circadian rhythm at both the mRNA and protein levels. Nocturnin encodes a deadenylase that is involved in the removal of polyA tails from mRNAs (Green et al., 2007). Nocturnin is not only directly involve in lipid absorption but also appears to be important in regulating unknown reduced lipid trafficking in the small intestine. The exact mechanism responsible for Nocturnin-mediated promotion of lipid secretion remains unknown. Nocturnin has also been confirmed to participate in adipogenesis, glucose homeostasis, osteogenesis and immune functions. Nocturnin in Per2^-/-^ knockout mice have normal circadian mechanisms, although they display resistance to HFD-induced obesity and hepatic steatosis, which indicates that Nocturnin is downstream of the core circadian clock (Stubblefield et al., 2018). During the daily circadian cycle and acute nutritional challenges, Nocturnin in Per2^-/-^ knockout mice have markedly elevated metabolism of cholesterol and TG (Stubblefield et al., 2012).

### Decs

DECs, which encode the bHLH transcription factors, can regulate the circadian rhythm and metabolism. DEC1 in Per2^-/-^ knockout mice show decreased lipid levels, reduced oxidative stress, and increased fibroblast growth factor 21 (FGF21) expression levels (Fujita et al., 2016). In DEC2 in Per2^-/-^ knockout mice livers, phosphorylation of AMP-activated protein kinase (pAMPK) is remarkably increased. Insulin and glucose can induce DEC1 and DEC2 expression, while under conditions of depleted glucose, the expression of both DEC1 and DEC2 is decreased (Sato et al., 2016). Liver X receptor (LXR) can induce DEC1 expression by binding its promoter. Blocking phosphoinositide 3-kinase, protein kinase C, or the mammalian target of rapamycin can also inhibit insulin-induced DEC1 and DEC2 expression. Both DEC1 and DEC2 can regulate adipogenesis by repressing the transcription of PPARγ. Furthermore, over-expressing DEC1 suppresses adipocyte differentiation. It has also been found that DEC1 can interact with the DNA-bound CCAAT/enhancer binding protein and repress PPARγ expression (Park and Park, 2012).

## The Circadian Clock Genes and the Progress of Nafld

The classic “two-hit hypothesis” proposes that the progression of NAFLD is initiated by hepatic fat accumulation (the first hit), and subsequent hits by a combination of oxidative stress, cytokines, bacterial endotoxin or stress at the level of the endoplasmic reticulum (ER) (the second hit). Recently, the coordinated interactions of autophagy and the host gut-microbiota has been identified as also representing additional biological insults (Nseir et al., 2014). We have already discussed above circadian clock genes that can induce hepatic steatosis. Moreover, studies have shown that circadian clock genes induce the progression of NAFLD (Froy, 2017).

Two investigations have suggested a protective effect of PER2 protein expression in acute liver injury and fibrosis. In the carbon tetrachloride-induced hepatitis model, Per2^-/-^ knockout mice progressed to a more severe form of hepatic fibrosis with hepatic stellate cell activation (Chen et al., 2010). In the cholestatic hepatitis model, lack of PER2 expression could also cause more severe fibrotic injury and the accumulation of extra cellular matrix (Chen et al., 2013).

In the context of a progression of liver tumorigenesis, growing evidence suggests that circadian clock genes play key roles in cell cycle regulation, checkpoint determination, genomic stability, and DNA repair. In Cry1/2 double knockout and Clock mutant mice models, *Wee1*, which monitors cell-cycle progression from the G2 to M transition, exhibits a disrupted circadian expression (Stevenson, 2017). NPAS2 can be overexpressed in hepatocellular carcinoma (HCC) and induces cell survival by promoting cell proliferation and inhibiting mitochondria-related intrinsic apoptosis both *in vitro* and *in vivo*. Transcriptionally upregulating CDC25A phosphatase can stimulate NPAS2 expression. Moreover, Bmal1 can heterodimerize with NPAS2, bind to the E-box element in the promoter region of CDC25A and participate in NPAS2-mediated tumor cell survival in HCC (Yuan et al., 2017). Per^-/-^ or Cry^-/-^ knockout mice can form fewer but larger HCCs that are first detected at the age of 50 weeks, while Alb^cre^; Bmal1^fl/fl^ mice form increased numbers but smaller HCCs that are first detected at the age of 70 weeks. Chronic jet lag can increase both the numbers and size of HCCs in Per^-/-^ or Cry^-/-^ knockout mice and the size of HCCs in Alb^cre^; Bmal1^fl/fl^ mice. Both sexes of mutant mice have an increased risk of HCC, while males show a comparatively greater risk of developing HCC than do females (Kettner et al., 2016).

## The Circadian Clock Genes and Nafld in Humans

Genomic variants in circadian clock genes are closely associated with hepatic steatosis, and predispose to NAFLD development. In humans, the clinical conditions of obesity, NAFLD and metabolic syndrome are associated with polymorphisms present in the Clock gene. Clock gene variants that include rs11932595 and rs6843722 show a close connection to NAFLD. A remarkable association is also found between the clinical or histologic spectrum of NAFLD and the presence of rs1554483, rs6843722, and rs6850524, and between the fibrosis score and the presence of rs1554483, rs6843722, and rs4864548.

Haplotypic association analyses show that Clock gene variant haplotype frequencies in NAFLD are quite different from those in controls (Sookoian et al., 2007). *Clock* rs3749474 is associated with total energy intake that might also be influenced by specific cytokine [e.g., monocyte chemotactic protein 1(MCP1), IL-6 and adiponectin] alterations (Ando et al., 2009). The *Clock* 3111T/C single-nucleotide polymorphism in women correlates with being overweight, the presence of circadian abnormalities and is characterized by “evening-type” subjects. Recognizing Clock type genotypes can help manage the causal roots of the metabolic problem (Bandín et al., 2013).

The B*mal*2 rs7958822 genotype shows a significant association with type 2 diabetes (T2DM) among obese Japanese individuals (Yamaguchi et al., 2015). The mRNA expression patterns of Bmal1, Per1, Per2, and Per3 are 24 h rhythmic and lower expression levels in the peripheral leucocytes of T2DM patients, while the transcriptional expression patterns are inversely correlated with HbA1c levels. Clock gene (Per2, Bmal1, and Cry1) expression patterns are sex dependent in human adipose tissues derived from morbidly obese subjects. In addition, the three clock genes are remarkably and negatively associated with the level of total cholesterol and low density lipoprotein (LDL) levels. Per2 expression in the visceral depot is inversely associated with waist circumference (Gomez-Abellan et al., 2008; Garaulet et al., 2010). The methylation status of CpG sites located in clock genes (Clock, Bmal1, and Per2) is associated with obesity, metabolic syndrome and weight loss. The differential methylation of a variety of CpG sites in Clock and Per2 indicates the success of weight-loss, especially in the context of Clock CPGs 5–6 (Milagro et al., 2012). The methylation of several CpGs in the PER3 is indicating the development of childhood obesity (Samblas et al., 2018).

In the Nocturnin gene, rs9684900 is closely related to the body mass index (BMI) among a study of 1, 510 non-diabetic Chinese subjects in Taiwan. Nocturnin mRNA levels in human abdominal adipose tissues are also elevated in obese as compared with non-obese subjects (Chang et al., 2013). Here, we only list a few closely related circadian clock genes, and indeed, we recognize that there are many other genes related to lipid metabolism, which are also controlled by circadian oscillators, which can react to the molecular networks of the circadian clock (Li et al., 2017).

In addition to circadian gene polymorphisms, feeding/fasting cycles, feeding time, sleep deprivation, and sleep quality all have prominent effects on circadian clocks. They can disrupt the circadian rhythms and interfere the metabolism. The optimal feeding time and the optimal sleep duration may decrease the risk of metabolism syndrome and NAFLD (Shetty et al., 2018).

## Conclusion

During the past several decades, an understanding of the circadian clock and metabolism has made significant advances. Moreover, environments that include cycles of rest and activity, feeding/fasting times and social stressors, have tremendous impacts on human physiology and metabolism (Angelousi et al., 2018). NAFLD, which is recognized as a lipid metabolic disease is closely connected with the circadian clock. Indeed, an increasing number of circadian rhythm studies have provided important insights that have enabled correlating expression of the circadian clock gene with metabolism in NAFLD within the holistic understanding of the involved molecular mechanisms. However, research investigators in the field of circadian metabolism are only beginning to understand the systems and mechanism, and clearly require additional experimentation to further broaden our comprehension of lipid metabolism in the liver (Wright et al., 2013; Sun et al., 2015). Clinical studies of circadian clock genes remain scarce in NAFLD patients. Hence, new insights that target key circadian clock genes with the intention of treating NAFLD may provide more effective strategies, pharmacological approaches, and improved guidance for specific nutrient components in the human diet.

## Author Contributions

DS designed the research hypotheses and objectives and wrote the manuscript. JC and JY performed detailed searches of the literature. JW and YH analyzed the data. GZ programmed and coordinated the writing. ZB critically analyzed and revised the manuscript.

## Conflict of Interest Statement

The authors declare that the research was conducted in the absence of any commercial or financial relationships that could be construed as a potential conflict of interest.
